# A pilot study of the cardiopulmonary effects in healthy volunteers after exposure to high levels of PM_2.5_ in a New York City subway station

**DOI:** 10.1186/s12989-024-00594-6

**Published:** 2024-10-08

**Authors:** David G. Luglio, Kayla Rae Farrell, Terry Gordon

**Affiliations:** 1 Department of Environmental Health Sciences, School of Public Health and Tropical Medicine, Tulane University, New Orleans, LA USA; 2 Division of Environmental Medicine, Grossman School of Medicine, New York University, New York, NY USA

**Keywords:** Subway, Air pollution, PM2.5, Cardiopulmonary, Spirometry, Oscillometry, Heart rate variability, Urban environment, Metals, Exposure, Blood pressure

## Abstract

**Background:**

Subway systems are becoming increasingly common worldwide transporting large populations in major cities. PM_2.5_ concentrations have been demonstrated to be exceptionally high when underground, however. Studies on the impact of subway PM exposure on cardiopulmonary health in the United States are limited.

**Methods:**

Healthy volunteers in New York City were exposed to a 2-h visit on the 9th Street Station platform on the Port Authority Trans-Hudson train system. Blood pressure, heart rate variability (HRV), spirometry, and forced impulse oscillometry were measured, and urine, blood spot, and nasal swab biosamples were collected for cytokine analysis at the end of the 2-h exposure period. These endpoints were compared against individual control measurements collected after 2-h in a “clean” control space. In addition to paired comparisons, mixed effects models with subject as a random effect were employed to investigate the effect of the PM_2.5_ concentrations and visit type (i.e., subway vs. control).

**Results:**

Mean PM_2.5_ concentrations on the platform and during the control visit were 293.6 ± 65.7 (SD) and 4.6 ± 1.9 µg/m^3^, respectively. There was no change in any of the health metrics, but there was a non-significant trend for SDNN to be lower after subway exposure compared to control exposure. Total symptomatic scores did increase post-subway exposure compared to reported values prior to exposure or after the control visit. No significant changes in cytokine concentrations in any specimen type were observed. Mixed-effects models mostly corroborated these paired comparisons.

**Conclusions:**

Acute exposures to PM on a subway platform do not cause measurable cardiopulmonary effects apart from reductions in HRV and increases in symptoms in healthy volunteers. These findings match other studies that found little to no changes in lung function and blood pressure after exposure in underground subway stations. Future work should still target potentially more vulnerable populations, such as individuals with asthma or those who spend increased time underground on the subway such as transit workers.

**Supplementary Information:**

The online version contains supplementary material available at 10.1186/s12989-024-00594-6.

## Background

In 2019, over 100 million people rode on a subway a day around the world [1]. Despite the effects of the SARS-CoV-2 pandemic on ridership, this number is expected to grow as more cities build underground transit systems and older systems expand their network. For example, prior to 2000, only four cities in mainland China had subway systems and, today, there are over 25. Moscow’s Metro system added a total of 31 new stations upon completion of its “Big Circle Line” in 2023. Other lines have been recently or are currently being added to well-established subway systems, such as the Elizabeth Line in London and the Purple Line in Washington, DC. Entirely new systems are being built in Honolulu and Abidjan, although they are mainly at the surface level or on elevated rails. New York City (NYC), alone, moves more than 1 billion people each year [2]. NYC has two major rails systems, the subway of the Metropolitan Transit Authority (MTA) and the Port Authority Trans-Hudson line (PATH) rail system. The latter connects Manhattan to suburban areas in New Jersey and had an annual ridership of over 50 million in 2023 [3].

The importance of metropolitan train systems cannot be overstated. They move large numbers of people throughout a city in a cheap and fast manner. The relative low-cost of these systems is crucial for individuals who are less economically advantaged, and who often must travel long distances for work. The economic benefits of these systems are intertwined with their ability to move both workers and consumers. Moreover, this reliance on public transportation helps to effectively minimize urban energy footprints by reducing personal vehicle traffic. Outdoor air quality is thus improved by a reduction in vehicular emissions [4, 5]. Lu et al. [4], estimated that opening a new subway reduced outdoor ambient PM_2.5_ concentrations by 18 µg/m^3^ in Chinese cities. Thus, subways improve air quality by reducing the reliance on cars, particularly during rush hours.

Some subway systems, however, are notorious for being “dirty”. This is especially true in NYC, where the presence of trash, dirt, grime, and large populations of rats visible on the platforms exemplifies this fact. In addition, the air quality in NYC subways has been demonstrated to be poor, harboring high concentrations of PM_2.5_ [6–10]. These findings corroborate results observed globally in subway systems [11–14]. What is increasingly concerning is that the composition of this PM is primarily heavy metals, particularly Fe [13, 15–20].

Little is known, however, about the potential effects this poor air quality has on the health of commuters and workers. Studies investigating the health impacts of subway exposure are few. Most of the early studies have been conducted in Stockholm [21–27], with a pilot study in New York [28]. More recent health research has occurred within the subway systems in Taipei, Beijing, London, and Paris [29–33]. Outside of symptomatic, heart rate variability (HRV), and a few molecular biomarker changes, significant effects on cardiovascular or pulmonary health have been limited. This includes measures of forced expiratory volumes. Oxylipin levels changed in healthy volunteers, but not in individuals with asthma after subway exposure [26]. Interestingly, Sauvain et al. [30] found that increased PM levels were associated with decreased acetate, lactate, and total NOx levels in exhaled breath condensate (EBC) during subway work shifts in transit workers. Overall, the lack of consistent evidence of adverse cardiopulmonary outcomes to date is reassuring for public health.

The lack of widespread studies is concerning, however, and confounds our ability to generalize from the available results for both commuters and transit workers. The latter are exposed for longer periods of time, and thus may be at greater risk, although they are expected to be generally less susceptible given the ‘healthy worker’ effect. Commuters, however, may include individuals who have pre-existing cardiopulmonary conditions, such as asthma, and may be at higher risk of adverse cardiopulmonary effects from subway PM exposure. Furthermore, the PM concentrations observed during the health studies conducted in Stockholm and Taiwan were lower than what is typically observed in NYC. As such, additional studies are warranted in a variety of subway systems.

The goal of this study was to assess the acute health risk to individuals after exposure to air pollution on subway platforms. Based upon previous work examining the adverse health effects associated with exposure to ambient PM_2.5_ [34, 35], various physiological parameters, such as lung function, heart rate metrics, and inflammatory markers, were assessed in commuters after an acute subway PM exposure. Comparisons of health effects between a subway PM exposure and a control-case baseline (i.e., a relatively “clean” environment) was done to determine the potential health risk of commuting via subway.

## Methods

To investigate the health consequences of subway commuter exposure, the cardiopulmonary effects of healthy volunteers were measured after a subway and a “clean” control location (i.e., location with low PM concentrations) exposure period of 2-h. NYU’s School of Medicine’s Institutional Review Board (IRB) approved all protocols and materials before this study commenced.

### Participant recruitment and exclusion criteria

Recruitment of research subjects was conducted through posting advertisements on Craigslist and through word-of-mouth methods. Subjects were encouraged to pass information to others in a snowball recruitment effort. Individuals involved in previous studies and who expressed consent to be contacted for future research were also potential contacts. Non-SARS-CoV-2 vaccinated individuals were barred from participation to reduce study complications and transmission risks. Other exclusion criteria included pre-existing cardiopulmonary conditions (e.g., asthma, COPD, heart disease), pregnancy, and/or report of current smoking, vaping, or use of other nicotine-containing products. Participants were between the ages of 18 and 55, able and willing to provide consent, and otherwise self-reported to be healthy (i.e., participants verbally stated that they consider themselves healthy). Recruitment was intended to generate an even biological sex distribution and racial/ethnic diversity to reflect the NYC population. All participants gave their informed consent prior to participation in this study.

### Exposure sites and protocols

Research subjects participated in two visits. The control visit, also referred to as the “clean” visit, assessed cardiopulmonary and symptom endpoints of each subject after a period of 2-h sitting in an office or courtyard space. These “clean” spaces represent areas of low PM levels with noise and temperature levels similar to those experienced in a subway station. The subway rail visit consisted of the same individuals being sitting on benches present (i.e., approximately 100 feet from the entrance) on the 9th Street Station platform on the PATH for a 2-h period. This rail system was selected because it represented some of the highest PM_2.5_ concentrations in previous studies [9, 10]. One study personnel was present with the participants during each visit; each visit had one to three participants. After the 2-h period, the same biospecimens and physiologic and symptom endpoints for both visits were taken at a laboratory space. The order of the visits (i.e., scheduling the control or subway visit as the initial visit) was randomized for each subject. The wash-out period between visits was at least 2-weeks, and there was no restriction on the participant’s lifestyle other than a request to not utilize the subway 24-h before each visit. Time between the two visits was kept to as close to 2 weeks as possible to limit effects of seasonal variability (i.e., visits occurred from summer to winter 2022). The timing of visits for each individual was kept consistent and mainly contained to the morning and evening rush hours (i.e., 8:00–10:00 AM and 3:00–6:00 PM, respectively).

PM and noise levels were measured for both the clean and subway visits of the study. The PM_2.5_ mass concentrations were measured on the subway platform gravimetrically. 37-mm diameter Teflon (Pall, Ann Arbor, Michigan) filter samples were collected using a 2.5 µm cut Personal Environmental Monitor (PEM) (SKC, Shoreview, Minnesota) and a calibrated Leland Legacy Pump (SKC, Inc.) operated at 10 L/min. Teflon filters were pre-conditioned to U.S. EPA-recommended relative humidity (RH) and temperature for a minimum of 24 h and the PM_2.5_ mass concentration was calculated through standard gravimetric analysis using a micro-balance (Model MT5, 1 µg readability, Mettler Toledo, Columbus, Ohio) performed in a temperature- and humidity-regulated weighing chamber (21 + 1 °C and 40 + 5% RH). Laboratory blank samples were used to correct for daily variation in the micro-balance analyses. A nephelometric-based DataRAM (pDR 1500, Thermo Fisher Scientific, Franklin, Massachusetts) was utilized to measure the PM_2.5_ concentrations during the clean visits. A sound-level meter (VLIKE, China), calibrated using a Teck (Tekcoplus Ltd), was used for all noise measurements.

In addition, participants were asked to complete a health questionnaire at the start and end of each visit. This questionnaire assessed adverse symptoms that the participants were experiencing. These symptoms included: cough, cough with phlegm, nasal irritation, sneezing, light headedness, fatigue, chest pain, shortness of breath, difficulty breathing, and headache, with backache as a control. Each symptom was rated on a scale from 1 to 10, with 10 being the most severe. Furthermore, the survey, a modified form utilized in previous studies [34], asked for demographic information and recent history about lifestyle and health. This included their regular transportation mode and time spent commuting.

### Cardiopulmonary measures

Following each exposure period, heart rate (HR), HR variability (HRV), blood pressure (BP), forced impulse oscillometry, and spirometry measurements were performed. In addition, nasal epithelial cell, urine, and blood samples were collected from every individual after the exposures for each visit.

Subjects wore a Polar H7 or H9 chest strap (Polar, Oulu, Finland) for a minimum of 15 min after arriving at the measurement location while seated. The data for an individual’s HR and RR intervals were recorded on Marco Altini’s HRV logger App (ASMA BV, Amsterdam, The Netherlands). Only the last 5 min of the 15-min period (i.e., when the individuals came to a resting condition) were used for analysis. Standard deviation of normal–normal intervals (SDNN), root mean square of successive differences (RMSSD), and the proportion of normal–normal intervals above 50 ms (pNN50) were derived from the RR interval data.

Systolic and diastolic blood pressure were measured with an Omron automated wrist monitor (Model BP629N, Omron Healthcare, Lake Forest, IL). The strap was placed on the left arm and operated with an open palm. Three measurements were taken at 1-min intervals and the last two measures were averaged and included in the analysis, as previously recommended [36].

In the forced impulse oscillometry measurements, pulmonary reactance and resistance were assessed with a Tremoflo C-100 Airwave Oscillometry System using American Thoracic Society (ATS) quality criteria (Thorasys, Montréal, Canada). Resistances in large and small airways and areas of reactance (R5, R5-20, and Ax values, respectively) were calculated as the mean of the 3 best trials with a coefficient of variation under 15%. Predicted values were calculated using the age, height, weight, gender, and race/ethnicity of the subject.

Forced expiratory volumes and vital capacity (FEV_1_ and FVC, respectively) were measured using an EasyOne spirometer (ndd Medical Technologies, Andover, MA) and ATS quality criteria. The best effort of a targeted set of 3 acceptable trials (ATS guidelines) was included in the analysis. Participants were coached through the process, and the correct procedure was first demonstrated prior to their attempts and feedback was given after each attempt. Predicted values were calculated using the age, height, gender, and race/ethnicity of the subjects.

### Biospecimen collection

Epithelial lining fluid was collected from both nares of each individual. Sterile saline was sprayed (100 µL, Amazon, Seattle, WA) into each nostril of the subject before Leukosorb filter strips (Cytiva, Marlborough, MA) were inserted [37]. A plastic clip was placed to clamp the nose for 2 min, after which the filter strips were removed, placed in microcentrifuge tubes (Fisher Scientific, Waltham, MA), and stored at − 20 °C until analysis.

Urine was collected at the end of each visit using a sterile polypropylene urine collection cup (Globe Scientific Company, Mahwah, NJ). The sample was aliquoted into 2 mL microcentrifuge tubes (Fisher Scientific), and any remainder was transferred to a 50 mL Falcon tube (Corning). Six microcentrifuge tube samples were centrifuged at 2,1130 RCF for 3 min to remove cellular debris and the supernatant was transferred to 1.5 mL microcentrifuge tubes (Fisher Scientific). All urine samples were stored at − 20 °C until analysis.

Dried blood spot samples were collected onto a Whatman 903 Protein Saver filter card (Cytiva) using an Unistik 2 or 3 Safety Lancet (Owen Mumford, Woodstock, Oxfordshire, UK). The filter card was allowed to air dry for at least 24-h (a maximum of 2 weeks) and stored at − 20 °C until analysis.

### Inflammatory cytokine analysis

All biospecimen samples were processed for cytokine protein analysis using the Meso Scale Discovery (MSD) V-PLEX Proinflammatory Panel 1 (human) Kit (Meso Scale Discovery, Natick, MA). This is a sandwich immunoassay, in which samples are added to a 96-well plate which has ten immobilized capture antibodies at the bottom at each well (i.e., IFN-γ, IL-1β, IL-2, IL-4, IL-6, IL-8, IL-10, IL-12p70, IL13, and TNF-α). After addition of the samples, electrochemiluminescent capture antibodies were added, followed by a “read” buffer, and then the plate was analyzed in an MSD plate reader (MESO QuickPlex SQ 120MM, Meso Scale Discovery).

Samples were eluted from the nasal strips after being soaked with 100 μL of a 1% BSA + 0.05% Triton X-100 in Dulbecco’s PBS (GIBCO/Thermo Fischer, Waltham, MD) and centrifuged at 21,130 RCF for 3 min. A hole, created using an 18-gauge needle, was first placed into the bottom of the microcentrifuge tube containing the nasal strip, which was then placed into a larger 2 mL tube (Fisher Scientific) for eluent collection during centrifugation. This nasal lining eluent was then added to the plate. Blood proteins were extracted from the dried blood spot filter cards. A six-mm hole punch was taken from fully saturated sections of the dried blood spots. For extraction, each punch was placed into a well of a 96 deep-well plate (USA Scientific, Ocala, FL) and immersed in 200 µL of PBS with 0.5% Tween 20 before shaking overnight at 4 °C at 5 rocking motions per minute. The supernatant was added to the MSD plate. Urine sample supernatants were added directly to the plate.

### Statistical analysis and mixed-effects model for cardiopulmonary endpoints

Means for each of the endpoints measured at the subway and clean visits were compared by paired t-tests, where data were matched by subject. Normality for each of the variables per group (i.e., clean vs. subway) was evaluated with the Shapiro–Wilk normality test. For endpoints that failed the normality test, the Wilcoxon signed rank exact test was employed (i.e., systolic BP, pNN50, R5, R5-20, Ax, symptoms).

Mixed-effects models were constructed to evaluate the influence on PM_2.5_ concentrations on the various health endpoints. Random effects included participant identity whereas visit location (i.e., categorical variable: clean = 0, subway = 1) and PM_2.5_ concentrations (continuous variable) were included as the fixed variable in alternative models. Predicted FEV1 (continuous), BMI (continuous), whether the participants had allergies (categorical: no = 0, yes = 1), and cumulative weekly time on the subway (continuous) were included as covariates. The slope coefficients represent the fractional change in an endpoint with increases in the unit value of each factor. Post-hoc Wald chi-square analysis was conducted on each of the covariates to determine whether its influence was significant. Linear mixed-effects models (LMM) were built using the BP, FEV_1_, FVC, R5, and pNN50 data. A generalized linear mixed-effects model (GLMM) was used for the SDNN, RMSSD, and Ax endpoints because they did not meet the assumptions of a LMM. These models were fitted with a Laplace Approximation with a Gaussian fitting distribution and a log or square-root link function. Mixed effects logistic regression was employed to analyze the total symptom score and R5-20 endpoints. Datapoints were assigned a “high” or “low” score based on whether the value of the endpoint variable was greater than or less than/equal to the median value in the data set (i.e., 15 for symptom score and 0.110 for R5-20). Odds ratios are presented per standard deviation increase in PM_2.5_ concentrations (i.e., across all visits; 155.7 µg/m^3^). All analyses were conducted in R [38] with the car [39], Lme4 [40], and performance [41] packages.

## Results

### Demographics of participants

Demographics for the participants are displayed in Table 1. There was a total of 28 participants included in the study. The study was evenly split between males and females, and individuals aged from 21 to 30 comprised 46% of the total participants. A portion of the participants reported having allergies, which were mainly to pollen and dust mites.

**Table 1:** Selected demographics and lifestyle characteristics of population

Characteristic	Number of participants	Percent of total
Gender		
Male	14	50
Female	14	50
Age		
18–20	1	4
21–30	13	46
31–40	6	21
41–50	4	14
51–55	4	14
Race/ethnicity		
Black	14	50
White	11	39
Asian	1	4
Mixed	2	7
Hispanic	7	25
Allergies (e.g., pollen, dust mites, pet dander, mold)	6	21

	Mean (± SD)	Median (IQR)
Height (cm)	172.8 ± 9.8	173 (13)
Weight (kg)	80.0 ± 20.9	79.5 (35)
BMI	26.5 ± 5.6	26.2 (9.0)
Predicted FEV1 (L)	3.65 ± 0.76	3.65 (0.94)
Predicted FVC (L)	4.39 ± 0.92	4.28 (1.09)

	Number of participants (% of total)	
	Control visit	Subway visit
Caffeine 3-h prior to visit	14 (50)	13 (46)
Frequently exposed to secondhand smoke 1-week prior to visit	5 (18)	3 (10)
Vegetarian diet 1-week prior to visit	3 (11)	5 (21)
Exposure to hookah 1-week prior to visit	0 (0)	1 (4)
Commute by train	25 (89)	23 (82)
Commute by bus/car	11 (39)	9 (32)
Commute by walking/biking	14 (50)	13 (46)

	Reported mean (± SD) before visit	
	Control visit	Subway visit
Frequency of subway riding per week	10.1 ± 4.6	9.6 ± 5.4
Estimated total time on subway each week (hours)	6.2 ± 5.9	5.5 ± 5.9

*SD* standard deviation, *IQR* interquartile range (3rd quartile–1st quartile)

The majority of participants in the study regularly commuted by subway train. Several of these individuals also reported traveling by bus, car, or walking as an alternative or as a mixed commute. Various participants changed their work and travel pattern between the two visits. In one extreme case, the individual’s train-use changed from 0 to 20 h per week between the two visits (first visit was on the subway). Overall, however, the mean frequency of use and the total time spent on a subway was similar before either visit (Table 1).

Approximately half of the individuals enrolled were overweight (i.e., BMI ≥ 25), with 8 of the 28 (i.e., 29%) being obese (i.e., BMI ≥ 30). In addition, some individuals were exposed to secondhand smoke within the week before their visits.

### PM and noise during control and subway visits

The mean PM_2.5_ concentration (± SD) measured on the 9th Street Station platform, the site of the subway exposures, was 293.6 ± 65.7 µg/m^3^, whereas the mean concentration at the control background site was 4.6 ± 1.9 µg/m^3^. Furthermore, the ranges of PM_2.5_ concentrations across the two sets of sites were 149.5–384.3 µg/m^3^ and 2.1–7.2 µg/m^3^, respectively. The general noise level in the subway was 65 dB with peaks occurring around 80–90 dB when a train was passing through the station, a common occurrence. In the courtyard of the control site, the sound level ranged from 65 to 70 dB, whereas in the office (i.e., during inclement weather), these readings dropped to 42–45 dB. Although temperatures were not recorded, the subway platform and the courtyard were relatively hot during the summer months and cool during the fall months.

### Self-reported symptoms of participants

**Fig. 1:**
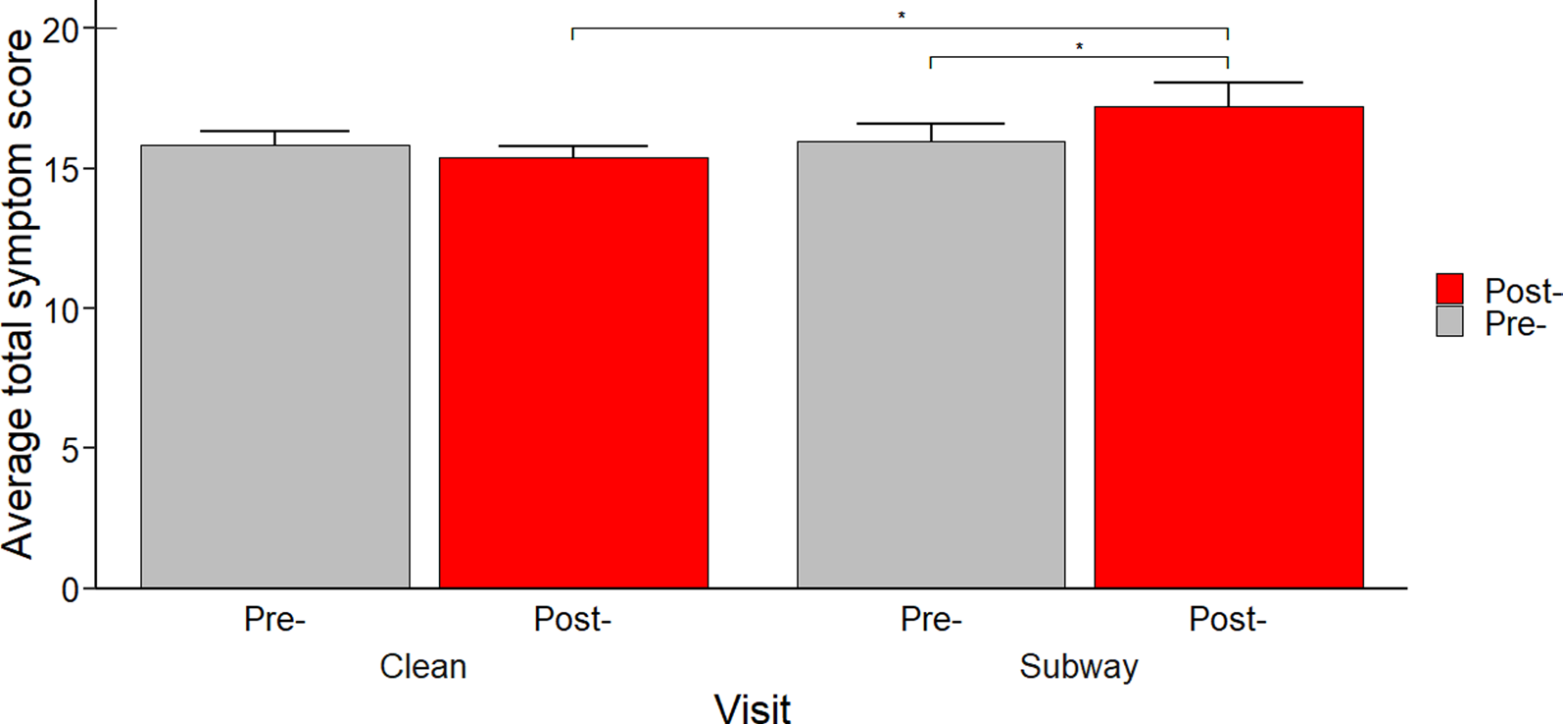
Total symptom scores before and after each 2-h “clean” and subway visit. Error bars indicate the standard error. The scale for the symptom scores is 1 to 10, with 10 being the most severe; the minimum total score for an individual was 15 and the highest is 150. *Indicates *p* value < 0.05, as determined by Wilcoxon signed rank tests (n = 27)

**Fig. 2:**
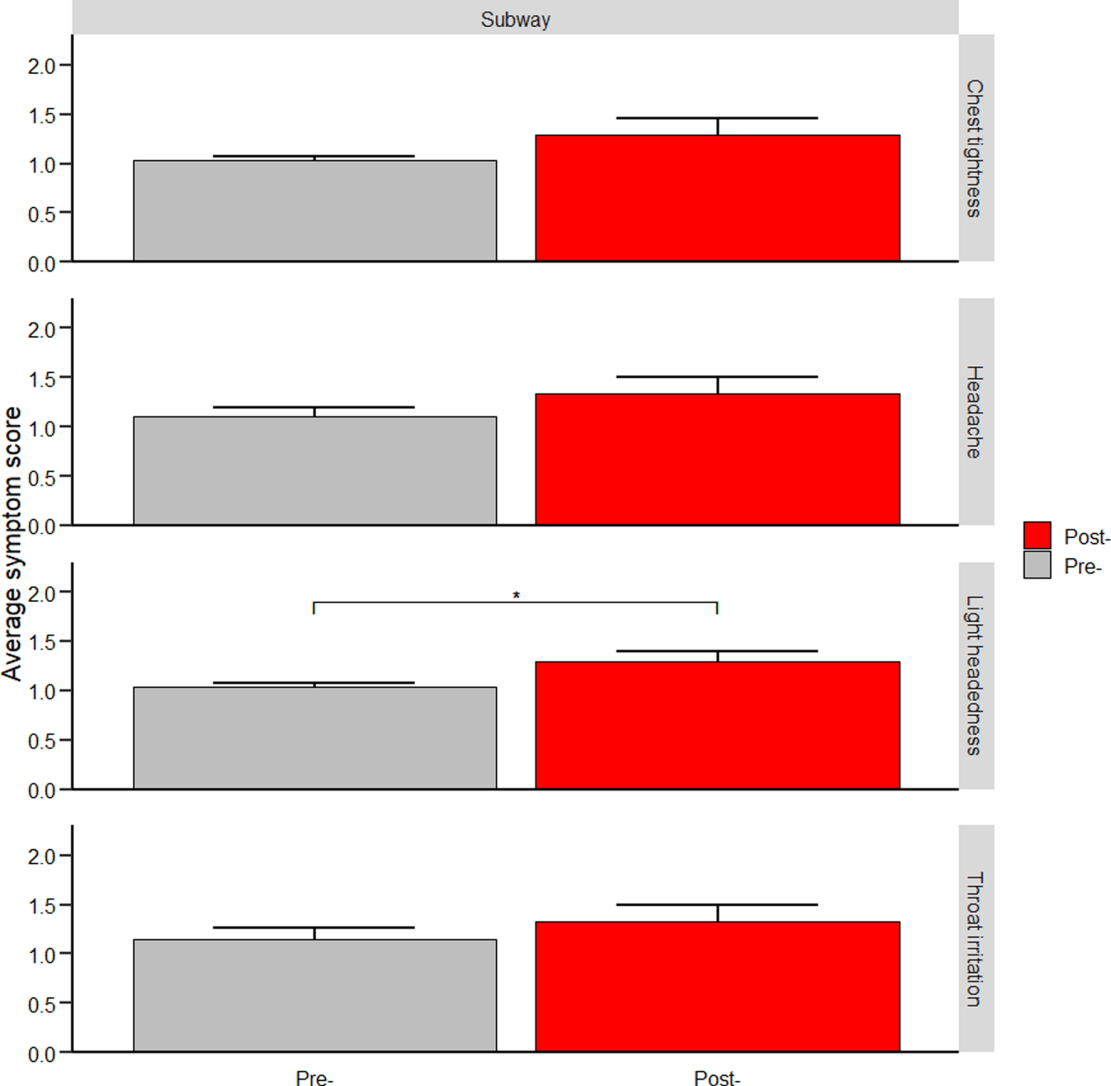
Severity of select symptoms before and after each 2-h subway visit. Error bars indicate the standard error. Each symptom is rated on a scale from 1 to 10, with 10 being the most severe. *Indicates *p* value < 0.05 as determined by Wilcoxon signed rank (n = 27)

Most participants reported few or no symptoms prior to both visits (i.e., average symptom score for each symptom was below 2 on a scale of 1–10; Table S1). Nasal congestion and back ache were the most common symptoms. The average total symptom scores (i.e., total of all symptom values excluding backache for an individual) are shown in Fig. 1 and individual scores in Table S1. Post-clean visit total scores were lower than pre-clean visit total scores (n = 27). On the other hand, total symptom scores were significantly increased post-subway visit compared to pre-subway visit. Additionally, total symptom scores were significantly higher post-subway visit than post-clean visit (n = 27; one participant was excluded due to missing post-subway visit data). Figure 2 presents the three individual symptoms showing the greatest change in severity. The only statistically significant individual symptom change, however, was an increase in light headedness after the 2-h subway session. Backache acting as a negative control (where no changes were expected to occur as result of air pollution exposure) showed no significant changes in its severity score pre- and post-visits. Backache was greater after the subway visit compared to after the control visit, however, and this difference was significant (*p* = 0.02).

### Physiological outcomes

As seen in Table 2, there was no significant effect of the exposure to subway PM on blood pressure, HR and HRV, spirometry, and forced impulse oscillometry.

**Table 2:** Results of physiological function tests post-exposures

Endpoint	Number of subjects	Control	Subway	*p* value
Systolic blood pressure	28	122.11 ± 3.51	121.64 ± 2.84	0.97^a^
Diastolic blood pressure	28	80.64 ± 2.73	78.36 ± 2.40	0.35
SDNN	16	54.53 ± 5.55	51.66 ± 4.67	0.53
RMSSD	16	45.40 ± 6.81	40.16 ± 5.00	0.33
pNN50	16	23.79 ± 5.39	18.56 ± 4.33	0.41^a^
FEV1	16	3.21 ± 0.22	3.31 ± 0.23	0.17
FVC	16	3.94 ± 0.28	3.94 ± 0.28	0.99
R5	17	3.35 ± 0.32	3.37 ± 0.41	0.55^a^
R5-20	17	0.40 ± 0.18	0.30 ± 0.18	0.35^a^
Ax	17	8.59 ± 3.26	7.97 ± 2.56	0.61^a^

All these measurements were collected after 2-h exposures in a control environment (i.e., courtyard or office) where PM levels were low, or in the subway environment (i.e., 9th Street Station on PATH in NYC). These numbers represent means ± standard error. Post-exposure values were compared with paired t-tests unless noted

^a^Groups were compared with a paired Wilcoxon signed rank exact test

### Cytokine production

To investigate the inflammatory potential of exposure to subway PM, ten cytokines were analyzed in urine, nasal epithelial cell, and dried blood spot samples. There was no significant difference in any cytokine concentration between the 2 exposure visits (Table S2).

### Mixed effects model

In addition to the statistical analyses presented above, a mixed effects model was constructed to test the effects of a variety of variables, including PM, whilst accounting for random effects that may differ from subject to subject. The results of the various mixed effects models are included in Tables S3–S8. Predicted FEV1 was a significant predictor for several endpoints, whereas BMI was a strong factor for both systolic, diastolic BP, and total symptom score. PM_2.5_ concentration and visit were significant factors for total symptom score and RMSSD, but not any other factor.

## Discussion

The presence of high levels of airborne particles in underground subway stations provides a unique exposure paradigm for millions of commuters and transit workers on a daily basis. Iron oxides and organic carbons are the main components of subway PM [10] and, thus, subway PM is quite dissimilar, in terms of composition, to ambient PM. Understanding the potential adverse health effects of these unique subway particles is therefore important and the present study evaluated the physiological and biochemical responses to an acute exposure to subway PM in healthy subjects. Overall, there was little change in heart and lung function of the participants enrolled in this study when comparing measurements at the end of the subway versus the control 2-h challenges. Overall symptomatic metrics, however, were significantly changed after the subway visit in comparison to the control visit.

Altogether, total symptom scores were higher post-subway visit compared to pre-subway visit. In addition, individual headache, light headedness, chest tightness and throat irritation scores increased post- versus pre-subway. An increase in backache, which should be unrelated to air pollution, was also observed. Thus, non-air pollution factors, such as sound levels or uncomfortable seating, could be influencing these symptomatic changes. Notably, the sound levels at the control site, located adjacent to a busy roadway in NYC, were similar to that on the underground subway platform except when peak subway station sound level increases were caused by a passing train. High noise levels could be tied to some symptoms, such as headache, as demonstrated by others [42, 43], but would not likely contribute to other symptoms like throat irritation. Ambient temperature could also influence symptoms or the perception of them, but this variable was not recorded in this study. The lack of chairs with a backrest in the subway could have contributed to the reported backache symptom. Changes in score post- to pre-clean visit were not consistent across these symptoms.

The logistic mixed effects models further support a “subway-exposure” effect. Visit location was found to be a significant factor for total post-visit symptom score alongside BMI. An increase in PM_2.5_ concentration, however, did not result in a significantly greater odds of a high symptom score although it did approach statistical significance (*p* = 0.06). Respiratory symptoms have previously been reported to have increased in volunteers on a Stockholm subway platform. It should be noted that the PM exposure levels in the Stockholm subway were much lower than the exposure levels encountered in the present study, yet they observed that upper respiratory symptoms increased for individuals with asthma [25] and lower airway symptoms increased for healthy volunteers [27].

As for cardiovascular outcomes, we did not observe any adverse changes associated with exposure to subway PM. BP, which has been shown to be affected by exposure to ambient PM concentrations [44], was not affected by the subway visit in the present study. This is potentially because the measurements of BP can be affected by various factors such as noise, stress, and activity levels [45–48]. Alternatively, the iron-laden subway PM may not affect the cardiovascular system as does ambient PM. In agreement with the pairwise comparisons above, PM_2.5_ concentrations and visit location were not significant factors for systolic or diastolic BP in the mixed effects model. Predicted FEV1 was a significant predictor for BP and many of the endpoints discussed below. It is a variable that reflects expected lung function based on physiologic/demographic characteristics (e.g., gender, age, height) that are unrelated to the exposure at hand. These characteristics inherently differ by individual and thus represent baseline differences between them. It appears that these differences not only explain some of the variability in the lung function measurements (across all individuals) but other cardiopulmonary endpoints.

There was some evidence of a decrease in HRV, although the observed pairwise comparisons were not statistically significant. The change in SDNN was the most consistent HRV parameter in terms of the directionality of change (i.e., decrease) and came close to statistical significance with the removal of an outlier (an individual who appeared to doze off; *p* = 0.07). On the other hand, the mixed effects model analysis determined that both visit location and PM_2.5_ concentration were significant factors for RMSSD. Interestingly, although a significant relationship was observed for this measure of HRV, it was not observed for the other two (i.e., SDNN, pNN50). In a study in Taipei, HRV (i.e., SDNN and RMSSD, post- vs. pre-commute) for individuals riding on the subway for an hour was higher than for those walking or using a bus or car [29]. Notably, the subway PM concentrations in that study were actually lower than the outside aboveground PM concentrations (22.3 ug/m^3^ vs. 42.1 ug/m^3^, respectively). On the other hand, evidence of a negative relationship between PM levels and HRV was observed in Beijing [32]. This previous study assessed whether the use of a respirator (i.e., to reduce PM exposure), headphones (i.e., to reduce noise), or both would affect HRV metrics and BP when on the subway. Higher SDNN and high frequency domain power (i.e., a variable not analyzed in the current study) were associated with the interventions compared to no interventions. When considering the non-intervention trials only (i.e., no respirator or headphones), PM_2.5_ concentrations were inversely related to HRV values. Our study’s sample size was relatively small, however, suggesting a larger study is needed to replicate these other studies.

Changes in HRV have gained importance in air pollution research and a decrease in HRV may be attributed to less heart rate adaptability. Thus, the trend noted in the present study might be considered an adverse outcome in older individuals. In fact, lower HRV has been associated with increased risk of all-cause mortality [49], mortality in the instance of a myocardial infarction [50], and of the development of cardiovascular disease [51], in particular the development of myocardial ischemia [52]. Many of these studies, however, focused on older individuals (i.e., > 45 years old), and it must be noted that the participants in the present subway study were generally young and healthy. Nevertheless, we postulate that exposure to subway PM may potentially lead to a negative outcome on HRV.

Furthermore, BP, spirometry, and oscillometry metrics were not significantly changed by the 2-h exposure to subway PM. This is consistent across the pairwise comparisons and mixed effects models, although effects of visit location and PM_2.5_ concentration did approach significance for FEV1 in the mixed effects models (*p* = 0.08 for both). In general, these results match other studies which have shown no change in spirometry from exposure to the air quality encountered in underground subway stations [22, 25, 27]. In addition to FEV1 and FVC, some of these studies measured peak expiratory flows (PEF), which also showed no change [22, 25, 27]. Together, these data suggest that acute exposures on the subway do not alter lung function.

The time frame of the collection of the biosamples for the cytokine measurements may have been inadequate in our investigation. All three types of biospecimens were collected from participants within an hour of leaving the subway or control site. Within this time frame, it can be reasonably expected that effects might be seen in the nasal, but not in the blood and urine samples. Yet, no statistically significant changes in cytokine levels were observed in any of the 3 types of biospecimen samples. Other studies have looked at cytokine and other molecule production in commuters and workers and, unlike in the present study, blood was collected several hours (i.e., 14 h in Nyström et al. [27] and Klepczyńska-Nyström et al. [25]; overnight in Bigert et al. [21]) after exposure. Cumulatively, however, these studies have not shown consistent inflammatory cytokine findings. Changes in cytokines in healthy and asthmatic volunteers, such as IL-6 and IL-8, were not observed post-subway exposure and neither were the blood clotting factors, plasminogen activator inhibitor-1 (PAI-1) and fibrinogen in these volunteers [25, 27] or C-reactive protein in workers [21]. Nevertheless, when comparing across different transit worker job categories, workers on the subway platforms had elevated levels of PAI-1 and CRP compared to train drivers and ticket office staff. Unfortunately, we did not collect pre-visit physiological and biosample data and, thus, day-to-day variability in these outcomes may limit our findings.

Our study design was set to emulate an extreme scenario, in which an individual is stuck on an underground subway platform for an extended period of time. The 2-h exposure may match, however, the accumulated total time spent in the subway system, daily, for some commuters. This setup may overestimate a typical commuter PM exposure in NYC, because on-platform PM concentrations are greater than on on-train PM concentrations [8, 10]. Importantly, we hypothesized that observable adverse health effects from an acute exposure to the poor air quality in underground subway stations would occur in our extreme exposure study design. Yet while increases in symptoms were associated with the subway exposures, general physiological and inflammatory indices of adverse health effects were not observed. This study, however, was an investigation of an acute exposure scenario, whereas, in reality, urban subway commuters are exposed repeatedly on a chronic basis. Most subway riders (e.g., over three million New Yorkers) use it every day, albeit for shorter periods of time than described in this study. Cumulative exposure over months and years may cause adverse health effects as have been seen in other settings of high PM concentrations [53–55]. This chronic exposure scenario was not studied here, and only briefly addressed elsewhere. Bigert et al. [23] saw no increases in myocardial infarctions in 304 underground train drivers compared to the public (i.e., 153,807 men aged 40–69 in Stockholm County). Likewise, Gustavsson et al. [24] saw no increase in lung cancer in these workers. There was no significant evidence for increased COPD cases or lung function decreases with long term PM_10_ exposure in Parisian subway workers [33], although they did find that longer durations of exposure (i.e., by year) was associated with FEV1 being less than the lower limit of normal. In addition, subway workers in London did not have increased rates of cardiovascular or non-infectious respiratory sick leave, compared to aboveground office workers, except when limited to doctor-certified cases [31]. Finally, it would be difficult to study chronic adverse effects of exposure to air pollution in underground subway commuters, as it would be quite difficult to identify a NYC-equivalent population which does not use the subway system.

### Limitations

Importantly, the study presented here only examined acute exposures on a single underground NYC subway platform. Most New Yorkers are not naïve to the subway, and many use it many times a week. Subway use thus likely results in a chronic exposure and it is unknown the level of each study participant’s PM exposure while commuting to the study sites in the present study. A high exposure commute immediately prior to study participation, for example, may have complicated the study of cardiopulmonary function and thus the observed lack of adverse health effects may have resulted because there was physiological adaptation to a chronic subway PM exposure. It is acknowledged that the participants may have used different means to get to each site (e.g., car vs. walk vs. bike) and this complexity of life in NYC could have affected the results.

Furthermore, the study sample size was limited, especially for the spirometry, oscillometry, and HRV tests. In terms of the former two, some function tests were discarded for lack of meeting quality standards. Despite ATS guidelines being followed, performance of spirometry is a learned maneuver which may take some time to perfect. This limited the strength and power of the results. With larger sample sizes, some subway exposure-cardiopulmonary endpoint effects may become significant, particularly the HRV outcomes. Moreover, the collection timeline of the blood and urine samples was soon after the exposure. Particles would not be expected to have been inhaled, deposited, entered the bloodstream, traveled to vulnerable tissues, caused damage and the release of cytokines, all within 1 h from the end of exposure. Thus, these biospecimens may have been collected too early. In addition, minor changes to improve participant comfort unrelated to the air pollution exposure is recommended (e.g., providing comfortable chairs in the subway). Finally, the inclusion of noise, temperature, and other potential confounders were not incorporated into the mixed-effect analysis due to the lack of complete data for each individual.

## Conclusions and future directions

Despite the high PM concentrations measured in underground subway stations relative to ambient concentrations [7–14], there have been few observed health effects reported to date [21–27, 29]. Overall, based on the results in this and other studies, the major adverse health outcomes of subway exposure are symptomatic changes. Cardiopulmonary effects have not been observed here in the Northeastern United States or in Stockholm. Although riding the subway may not have acute harmful health effects, chronic exposure scenarios should be investigated in terms of daily commuting and transit worker exposures. Workers spend more time underground than a typical commuter, and thus have a higher cumulative exposure to subway PM. Therefore, investigations into worker health is highly warranted. The use of employee medical records would provide valuable information, particularly in regard to studying a chronic exposure effect.

It is also important to note that the cardiopulmonary work conducted in this study involved only healthy participants. Individuals with pre-existing cardiopulmonary conditions, such as asthma or COPD, are expected to be at higher risk. As demonstrated in other settings, the effect of a high PM acute exposure would be especially concerning for individuals with asthma [56, 57], who may experience acute exacerbations. It is important to protect as large a proportion of the population as possible, and so, efforts should be undertaken to evaluate the risk to health in susceptible individuals.

## Supplementary Information

Supplementary Material 1.

## Data Availability

The datasets analyzed during the current study are available from the corresponding author on reasonable request. All subject data will be de-identified when shared.
