# Supplementary material for: A pilot study of the cardiopulmonary effects in healthy volunteers after exposure to high levels of PM2.5 in a New York City subway station
Source: Part Fibre Toxicol. 2024 Oct 8;21:42. doi: 10.1186/s12989-024-00594-6 (PMC11460011; doi:10.1186/s12989-024-00594-6)
Supplement: Supplementary file 1 — Supplementary Material 1. [file 12989_2024_594_MOESM1_ESM.docx]

Table S1 – Average symptom scores of participants pre- and post-visits

|  | Clean (SE) |  |  | Subway (SE) |  |  |
| --- | --- | --- | --- | --- | --- | --- |
| Symptom | Pre- | Post- | Change | Pre- | Post- | Change |
| Shortness of breath | 1.00 (0) | 1.11 (0.11) | 0.11 (0.08) | 1.15 (0.07) | 1.25 (0.10) | 0.11 (0.10) |
| Cough | 1.07 (0.05) | 1.07 (0.05) | 0 (0) | 1.04 (0.04) | 1.18 (0.11) | 0.15 (0.12) |
| Cough with phlegm | 1.07 (0.05) | 1.07 (0.05) | 0 (0) | 1.15 (0.15) | 1.19 (0.15) | 0.04 (0.04) |
| Wheeze | 1.00 (0) | 1.00 (0) | 0 (0) | 1.00 (0) | 1.00 (0) | 0 (0) |
| Difficulty breathing | 1.07 (0.05) | 1.07 (0.05) | 0 (0.05) | 1.07 (0.07) | 1.11 (0.08) | 0.04 (0.04) |
| Throat irritation | 1.15 (0.11) | 1.22 (0.15) | 0.07 (0.05) | 1.15 (0.12) | 1.33 (0.16) | 0.19 (0.10) |
| Chest tightness | 1.15 (0.10) | 1.07 (0.05) | -0.07 (0.12) | 1.04 (0.04) | 1.30 (0.17) | 0.26 (0.17) |
| Nasal irritation | 1.00 (0) | 1.04 (0.04) | 0.04 (0.04) | 1.27 (0.26) | 1.44 (0.27) | 0.07 (0.07) |
| Nasal congestion | 1.26 (0.10) | 1.19 (0.08) | -0.07 (0.07) | 1.56 (0.21) | 1.41 (0.19) | -0.15 (0.16) |
| Runny nose | 1.3 (0.12) | 1.07 (0.05) | -0.26 (0.11) | 1.07 (0.05) | 1.07 (0.05) | 0 (0.05) |
| Eye irritation | 1.30 (0.17) | 1.19 (0.11) | -0.11 (0.18) | 1.22 (0.22) | 1.22 (0.22) | 0 (0) |
| Headache | 1.30 (0.14) | 1.07 (0.05) | -0.22 (0.13) | 1.11 (0.08) | 1.33 (0.17) | 0.22 (0.11) |
| Nausea | 1.04 (0.04) | 1.04 (0.04) | 0 (0.05) | 1.00 (0) | 1.07 (0.05) | 0.07 (0.05) |
| Light headedness | 1.11 (0.06) | 1.15 (0.09) | 0.04 (0.11) | 1.04 (0.04)^a^ | 1.30 (0.10)^a^ | 0.26 (0.11) |
| Total symptom score | 15.85 (0.49) | 15.37 (0.42)^b^ | -0.48 (0.39) | 15.96 (0.64)^a^ | 17.22 (0.82)^ab^ | 1.26 (0.52) |
| Back ache | 1.30 (0.20) | 1.26 (0.19)^b^ | -0.04 (0.06) | 1.63 (0.3) | 1.74(0.30)^b^ | 0.11 (0.08) |

Participants were asked to rate each of these symptoms from 1 – 10, with 10 being the most severe before and after each 2-hour exposure period for both visits. Change represents the difference between the Post- and Pre- values (i.e., Post minus Pre) for either visit. Total symptom score excludes backache which is a control.

SE – standard error

^a^ -- p-value < 0.05; comparison: Subway Pre-, Subway Post-

^b^ -- p-value < 0.05; comparison: Clean Post-, Subway Post-

Groups were compared with a paired Wilcoxon signed rank exact test

Table S2 – Cytokine concentrations in blood, urine, and nasal swabs of participants after a 2-hour subway exposure

|  |  |  | Concentration (pg/mL) |
| --- | --- | --- | --- |
| Cytokine | Sample source | Visit | Mean +/- SE |
| IFN-γ | Urine | Clean | 1.22 +/- 0.21 |
|  |  | Subway | 1.46 +/- 0.20 |
|  | Nasal swab | Clean | 14.26 +/- 6.59 |
|  |  | Subway | 40.90 +/- 20.32 |
|  | Blood | Clean | 0.64 +/- 0.41 |
|  |  | Subway | 0.04 +/- 0.46 |
| IL-10 | Urine | Clean | 0.18 +/- 0.03 |
|  |  | Subway | 0.19 +/- 0.02 |
|  | Nasal swab | Clean | 1.97 +/- 0.61 |
|  |  | Subway | 4.13 +/- 1.61 |
|  | Blood | Clean | 0.05 +/- 0.04 |
|  |  | Subway | 0.06 +/- 0.04 |
| IL-12p70 | Urine | Clean | 0.33 +/- 0.05 |
|  |  | Subway | 0.39 +/- 0.05 |
|  | Nasal swab | Clean | 3.10 +/- 1.64 |
|  |  | Subway | 2.42 +/- 0.92 |
|  | Blood | Clean | 0.13 +/- 0.06 |
|  |  | Subway | 0.09 +/- 0.06 |
| IL-13 | Urine | Clean | 3.28 +/- 0.36 |
|  |  | Subway | 3.72 +/- 0.39 |
|  | Nasal swab | Clean | 25.32 +/- 3.71 |
|  |  | Subway | 25.46 +/- 3.26 |
|  | Blood | Clean | 0.82 +/- 0.22 |
|  |  | Subway | 0.82 +/- 0.22 |
| IL-1ß | Urine | Clean | 0.82 +/- 0.30 |
|  |  | Subway | 0.48 +/- 0.10 |
|  | Nasal swab | Clean | 86.55 +/- 21.15 |
|  |  | Subway | 166.18 +/- 50.03 |
|  | Blood | Clean | 1.19 +/- 0.58 |
|  |  | Subway | 0.92 +/- 0.29 |
| IL-2 | Urine | Clean | 0.64 +/- 0.07 |
|  |  | Subway | 0.68 +/- 0.06 |
|  | Nasal swab | Clean | 9.39 +/- 6.03 |
|  |  | Subway | 2.88 +/- 0.49 |
|  | Blood | Clean | 0.12 +/- 0.07 |
|  |  | Subway | 0.09 +/- 0.05 |
| IL-4 | Urine | Clean | 0.08 +/- 0.01 |
|  |  | Subway | 0.10 +/- 0.01 |
|  | Nasal swab | Clean | 0.43 +/- 0.12 |
|  |  | Subway | 1.37 +/- 0.74 |
|  | Blood | Clean | 0.05 +/- 0.01 |
|  |  | Subway | 0.03 +/- 0.02 |
| IL-6 | Urine | Clean | 0.64 +/- 0.08 |
|  |  | Subway | 0.66 +/- 0.07 |
|  | Nasal swab | Clean | 44.79 +/- 16.24 |
|  |  | Subway | 65.03 +/- 18.13 |
|  | Blood | Clean | 0.20 +/- 0.10 |
|  |  | Subway | 0.26 +/- 0.03 |
| IL-8 | Urine | Clean | 50.93 +/- 30.85 |
|  |  | Subway | 30.01 +/- 14.06 |
|  | Nasal swab | Clean | 1053.70 +/- 82.83 |
|  |  | Subway | 1028.22 +/- 94.96 |
|  | Blood | Clean | 5.17 +/- 1.33 |
|  |  | Subway | 4.30 +/- 0.92 |
| TNF-α | Urine | Clean | 0.46 +/- 0.07 |
|  |  | Subway | 0.50 +/- 0.07 |
|  | Nasal swab | Clean | 8.26 +/- 2.0 |
|  |  | Subway | 9.81 +/- 1.74 |
|  | Blood | Clean | 0.28 +/- 0.12 |
|  |  | Subway | 0.16 +/- 0.07 |

There were 28 urine and 27 nasal swab samples for the clean and subway visits. Whereas there were only 5 blood samples per cytokine per visit. There were no significant differences between the clean and subway visits for any of the cytokines.

Table S3 – Linear mixed effects models of cardiopulmonary endpoints with visit-type as a fixed effect

| Model | Systolic blood pressure | | |
| --- | --- | --- | --- |
| **Predictors** | **Estimates** | **CI** | **p-value** |
| Visit [Subway] | -0.39 | (-5.39, 4.61) | 0.878 |
| BMI | 2.03 | (1.40, 2.66) | <0.001* |
| Allergies [Yes] | -16.41 | (-24.89, -7.92) | <0.001* |
| Predicted FEV1 (L/s) | -6.35 | (-10.88, -1.81) | 0.006* |
| Estimated time on subway (hours) | 0.12 | (-0.41, 0.65) | 0.660 |
| **Random effects** |  |  |  |
| σ^2^ | 90.75 |  |  |
| τ_00_ _Subject_ | 34.46 |  |  |
| ICC | 0.28 |  |  |
| N_Subject_ | 28 |  |  |
| Observations | 56 |  |  |
| Marginal R^2^/Conditional R^2^ | 0.55 / 0.68 |  |  |
| Model | Diastolic blood pressure | | |
| **Predictors** | **Estimates** | **CI** | **p-value** |
| Visit [Subway] | -2.17 | (-6.75, 2.41) | 0.353 |
| BMI | 1.42 | (0.87, 1.96) | <0.001* |
| Allergies [Yes] | -7.12 | (-14.46, 0.22) | 0.057 |
| Predicted FEV1 (L/s) | -6.18 | (-10.11, -2.26) | 0.002* |
| Estimated time on subway (hours) | 0.16 | (-0.30, 0.63) | 0.493 |
| **Random effects** |  |  |  |
| σ^2^ | 76.12 |  |  |
| τ_00_ _Subject_ | 21.71 |  |  |
| ICC | 0.22 |  |  |
| N_Subject_ | 28 |  |  |
| Observations | 56 |  |  |
| Marginal R^2^/Conditional R^2^ | 0.46 / 0.58 |  |  |
| Model | FEV1 | | |
| **Predictors** | **Estimates** | **CI** | **p-value** |
| Visit [Subway] | 0.13 | (-0.01, 0.28) | 0.076 |
| BMI | 0.03 | (-0.02, 0.07) | 0.271 |
| Allergies [Yes] | -0.31 | (-0.82, 0.19) | 0.226 |
| Predicted FEV1 (L/s) | 1.04 | (0.75, 1.33) | <0.001* |
| Estimated time on subway (hours) | 0.01 | (-0.01, 0.03) | 0.342 |
| **Random effects** |  |  |  |
| σ^2^ | 0.04 |  |  |
| τ_00_ _Subject_ | 0.13 |  |  |
| ICC | 0.77 |  |  |
| N_Subject_ | 16 |  |  |
| Observations | 32 |  |  |
| Marginal R^2^/Conditional R^2^ | 0.78 / 0.95 |  |  |
| Model | FVC |  |  |
| **Predictors** | **Estimates** | **CI** | **p-value** |
| Visit [Subway] | 0.02 | (-0.15, 0.19) | 0.840 |
| BMI | 0.03 | (-0.03, 0.09) | 0.381 |
| Allergies [Yes] | -0.26 | (-0.94, 0.42) | 0.447 |
| Predicted FEV1 (L/s) | 1.23 | (0.84, 1.61) | <0.001* |
| Estimated time on subway (hours) | 0.01 | (-0.02, 0.03) | 0.582 |
| **Random effects** |  |  |  |
| σ^2^ | 0.05 |  |  |
| τ_00_ _Subject_ | 0.26 |  |  |
| ICC | 0.83 |  |  |
| N_Subject_ | 16 |  |  |
| Observations | 32 |  |  |
| Marginal R^2^/Conditional R^2^ | 0.73 / 0.96 |  |  |
| Model | R5 |  |  |
| **Predictors** | **Estimates** | **CI** | **p-value** |
| Visit [Subway] | 0.04 | (-0.34, 0.42) | 0.839 |
| BMI | 0.08 | (-0.01, 0.16) | 0.068 |
| Allergies [Yes] | 0.88 | (-0.05, 1.81) | 0.065 |
| Predicted FEV1 (L/s) | -1.12 | (-1.67, -0.56) | <0.001* |
| Estimated time on subway (hours) | 0.01 | (-0.04, 0.06) | 0.639 |
| **Random effects** |  |  |  |
| σ^2^ | 0.29 |  |  |
| τ_00_ _Subject_ | 0.41 |  |  |
| ICC | 0.59 |  |  |
| N_Subject_ | 17 |  |  |
| Observations | 34 |  |  |
| Marginal R^2^/Conditional R^2^ | 0.68 / 0.87 |  |  |
| Model | pNN50 |  |  |
| **Predictors** | **Estimates** | **CI** | **p-value** |
| Visit [Subway] | -4.86 | (-12.91, 3.20) | 0.237 |
| BMI | 0.87 | (-0.53, 2.28) | 0.224 |
| Allergies [Yes] | -17.90 | (-35.56, -0.24) | 0.047* |
| Predicted FEV1 (L/s) | 12.76 | (3.07, 22.44) | 0.010* |
| Estimated time on subway (hours) | 0.19 | (-0.84, 1.23) | 0.716 |
| **Random effects** |  |  |  |
| σ^2^ | 126.7 |  |  |
| τ_00_ _Subject_ | 120.7 |  |  |
| ICC | 0.49 |  |  |
| N_Subject_ | 16 |  |  |
| Observations | 32 |  |  |
| Marginal R^2^/Conditional R^2^ | 0.32 / 0.65 |  |  |

Subjects were included as the random effect. Fixed effect covariates were visit-type BMI, preexisting allergies (i.e., pollen, dust, mold, etc.), predicted FEV1, PM_2.5_ concentration, total estimated time on subway per week. Wald chi-square tests were employed to determine the significance of the influence of each of the fixed effects variables. * indicates when p-values < 0.05. ICC: intraclass correlation

Table S4 – Linear mixed effects models of cardiopulmonary endpoints with PM_2.5_ concentration as a fixed effect

| Model | Systolic blood pressure | | |
| --- | --- | --- | --- |
| **Predictors** | **Estimates** | **CI** | **p-value** |
| PM_2.5_ concentration (µg/m^3^) | -0.01 | (-0.02, 0.01) | 0.561 |
| BMI | 2.04 | (1.41, 2.66) | <0.001* |
| Allergies [Yes] | -16.45 | (-24.90, -8.00) | <0.001* |
| Predicted FEV1 (L/s) | -6.38 | (-10.90, -1.86) | 0.005* |
| Estimated time on subway (hours) | 0.11 | (-0.42, 0.63) | 0.697 |
| **Random effects** |  |  |  |
| σ^2^ | 90.46 |  |  |
| τ_00_ _Subject_ | 33.97 |  |  |
| ICC | 0.27 |  |  |
| N_Subject_ | 28 |  |  |
| Observations | 56 |  |  |
| Marginal R^2^/Conditional R^2^ | 0.55 / 0.68 |  |  |
| Model | Diastolic blood pressure | | |
| **Predictors** | **Estimates** | **CI** | **p-value** |
| PM_2.5_ concentration (µg/m^3^) | -0.01 | (-0.02, 0.01) | 0.257 |
| BMI | 1.43 | (0.89, 1.97) | <0.001* |
| Allergies [Yes] | -7.21 | (-14.52, 0.11) | 0.054 |
| Predicted FEV1 | -6.26 | (-10.18, -2.35) | 0.002* |
| Estimated time on subway | 0.15 | (-0.32, 0.62) | 0.522 |
| **Random effects** |  |  |  |
| σ^2^ | 75.52 |  |  |
| τ_00_ _Subject_ | 21.60 |  |  |
| ICC | 0.22 |  |  |
| N_Subject_ | 28 |  |  |
| Observations | 56 |  |  |
| Marginal R^2^/Conditional R^2^ | 0.46 / 0.58 |  |  |
| Model | FEV1 | | |
| **Predictors** | **Estimates** | **CI** | **p-value** |
| PM_2.5_ concentration (µg/m^3^) | 0.0004 | (-0.0004, 0.0009) | 0.075 |
| BMI | 0.03 | (-0.02, 0.07) | 0.262 |
| Allergies [Yes] | -0.30 | (-0.80, 0.12) | 0.233 |
| Predicted FEV1 (L/s) | 1.05 | (0.76, 1.33) | <0.001* |
| Estimated time on subway (hours) | 0.012 | (-1.099, 0.035) | 0.309 |
| **Random effects** |  |  |  |
| σ^2^ | 0.04 |  |  |
| τ_00_ _Subject_ | 0.13 |  |  |
| ICC | 0.76 |  |  |
| N_Subject_ | 16 |  |  |
| Observations | 32 |  |  |
| Marginal R^2^/Conditional R^2^ | 0.79 / 0.95 |  |  |
| Model | FVC |  |  |
| **Predictors** | **Estimates** | **CI** | **p-value** |
| PM_2.5_ concentration (µg/m^3^) | 0.000005 | (-0.0005, 0.0006) | 0.985 |
| BMI | 0.03 | (-0.03, 0.09) | 0.377 |
| Allergies [Yes] | -0.27 | (-0.95, 0.41) | 0.439 |
| Predicted FEV1 (L/s) | 1.23 | (0.84, 1.61) | <0.001* |
| Estimated time on subway (hours) | 0.01 | (-0.02, 0.03) | 0.639 |
| **Random effects** |  |  |  |
| σ^2^ | 0.05 |  |  |
| τ_00_ _Subject_ | 0.25 |  |  |
| ICC | 0.83 |  |  |
| N_Subject_ | 16 |  |  |
| Observations | 32 |  |  |
| Marginal R^2^/Conditional R^2^ | 0.73 / 0.96 |  |  |
| Model | R5 |  |  |
| **Predictors** | **Estimates** | **CI** | **p-value** |
| PM_2.5_ concentration (µg/m^3^) | 0.0002 | (-0.001, 0.001) | 0.729 |
| BMI | 0.08 | (-0.01, 0.16) | 0.070 |
| Allergies [Yes] | 0.88 | (-0.05, 1.82) | 0.064 |
| Predicted FEV1 (L/s) | -1.11 | (-1.67, -0.56) | <0.001* |
| Estimated time on subway (hours) | 0.01 | (-0.04, 0.06) | 0.608 |
| **Random effects** |  |  |  |
| σ^2^ | 0.29 |  |  |
| τ_00_ _Subject_ | 0.42 |  |  |
| ICC | 0.59 |  |  |
| N_Subject_ | 17 |  |  |
| Observations | 34 |  |  |
| Marginal R^2^/Conditional R^2^ | 0.68 / 0.87 |  |  |
| Model | pNN50 |  |  |
| **Predictors** | **Estimates** | **CI** | **p-value** |
| PM_2.5_ concentration (µg/m^3^) | -0.01 | (-0.04, 0.01) | 0.312 |
| BMI | 0.87 | (-0.55, 2.29) | 0.229 |
| Allergies [Yes] | -17.96 | (-35.79, -0.13) | 0.048* |
| Predicted FEV1 (L/s) | 12.51 | (2.74, 22.28) | 0.012* |
| Estimated time on subway (hours) | 0.19 | (-0.86, 1.24) | 0.720 |
| **Random effects** |  |  |  |
| σ^2^ | 127.2 |  |  |
| τ_00_ _Subject_ | 124.0 |  |  |
| ICC | 0.49 |  |  |
| N_Subject_ | 16 |  |  |
| Observations | 32 |  |  |
| Marginal R^2^/Conditional R^2^ | 0.32 / 0.65 |  |  |

Subjects were included as the random effect. Fixed effect covariates were visit-type BMI, preexisting allergies (i.e., pollen, dust, mold, etc.), predicted FEV1, PM_2.5_ concentration, total estimated time on subway per week. Wald chi-square tests were employed to determine the significance of the influence of each of the fixed effects variables. * indicates when p-values < 0.05. ICC: intraclass correlation

Table S5 – Generalized mixed effects models of cardiopulmonary endpoints with visit-type as a fixed effect

| Model | SDNN | Link: square-root | |
| --- | --- | --- | --- |
| **Predictors** | **Estimates** | **CI** | **p-value** |
| Visit [Subway] | -0.25 | (-0.67, 0.18) | 0.257 |
| BMI | 0.08 | (-0.01, 0.17) | 0.069 |
| Allergies [Yes] | -0.74 | (-1.85, 0.37) | 0.189 |
| Predicted FEV1 (L/s) | 1.43 | (0.82, 2.03) | <0.001* |
| Estimated time on subway (hours) | -0.01 | (-0.07, 0.05) | 0.748 |
| **Random effects** |  |  |  |
| σ^2^ | 72.11 |  |  |
| τ_00_ _Subject_ | 51.81 |  |  |
| ICC | 0.42 |  |  |
| N_Subject_ | 16 |  |  |
| Observations | 32 |  |  |
| Marginal R^2^/Conditional R^2^ | 0.01 / 0.42 |  |  |
| Model | RMSSD | | |
| **Predictors** | **Estimates** | **CI** | **p-value** |
| Visit [Subway] | -0.56 | (-1.11, -0.01) | 0.045* |
| BMI | 0.11 | (-0.02, 0.24) | 0.107 |
| Allergies [Yes] | -1.45 | (-3.09, 0.19) | 0.083 |
| Predicted FEV1 (L/s) | 1.53 | (0.64, 2.43) | <0.001* |
| Estimated time on subway (hours) | -0.02 | (-0.09, 0.06) | 0.617 |
| **Random effects** |  |  |  |
| σ^2^ | 93.72 |  |  |
| τ_00_ _Subject_ | 147.98 |  |  |
| ICC | 0.61 |  |  |
| N_Subject_ | 16 |  |  |
| Observations | 32 |  |  |
| Marginal R^2^/Conditional R^2^ | 0.01 / 0.61 |  |  |
| Model | Ax | Link: log |  |
| **Predictors** | **Estimates** | **CI** | **p-value** |
| Visit [Subway] | 0.31 | (-0.24, 0.86) | 0.269 |
| BMI | 0.11 | (-0.01, 0.23) | 0.079 |
| Allergies [Yes] | 0.51 | (-0.85, 1.86) | 0.462 |
| Predicted FEV1 (L/s) | -0.10 | (-1.00, 0.81) | 0.836 |
| Estimated time on subway (hours) | -0.15 | (-0.31, -0.0004) | 0.049* |
| **Random effects** |  |  |  |
| σ^2^ | 12.12 |  |  |
| τ_00_ _Subject_ | 13.40 |  |  |
| ICC | 0.53 |  |  |
| N_Subject_ | 17 |  |  |
| Observations | 34 |  |  |
| Marginal R^2^/Conditional R^2^ | 0.06 / 0.55 |  |  |

Subjects were included as the random effect. Fixed effect covariates were visit-type BMI, preexisting allergies (i.e., pollen, dust, mold, etc.), predicted FEV1, PM_2.5_ concentration, total estimated time on subway per week. Wald chi-square tests were employed to determine the significance of the influence of each of the fixed effects variables. * indicates when p-values < 0.05. ICC: intraclass correlation

Table S6 – Generalized mixed effects models of cardiopulmonary endpoints with PM_2.5_ concentration as a fixed effect

| Model | SDNN | Link = square-root | |
| --- | --- | --- | --- |
| **Predictors** | **Estimates** | **CI** | **p-value** |
| PM_2.5_ concentration (µg/m^3^) | -0.001 | (-0.002, 0.0002) | 0.120 |
| BMI | 0.08 | (-0.01, 0.18) | 0.072 |
| Allergies [Yes] | -0.750 | (-1.90, 0.40) | 0.201 |
| Predicted FEV1 (L/s) | 1.43 | (0.80, 2.06) | <0.001* |
| Estimated time on subway (hours) | -0.02 | (-0.08, 0.04) | 0.525 |
| **Random effects** |  |  |  |
| σ^2^ | 67.37 |  |  |
| τ_00_ _Subject_ | 52.26 |  |  |
| ICC | 0.45 |  |  |
| N_Subject_ | 16 |  |  |
| Observations | 32 |  |  |
| Marginal R^2^/Conditional R^2^ | 0.01 / 0.44 |  |  |
| Model | RMSSD | | |
| **Predictors** | **Estimates** | **CI** | **p-value** |
| PM_2.5_ concentration (µg/m^3^) | -0.001 | (-0.001, -0.0001) | 0.013* |
| BMI | 0.04 | (-0.01, 0.08) | 0.083 |
| Allergies [Yes] | -0.46 | (-1.01, 0.09) | 0.104 |
| Predicted FEV1 | 0.55 | (0.25, 0.85) | <0.001* |
| Estimated time on subway | -0.01 | (-0.03, 0.01) | 0.237 |
| **Random effects** |  |  |  |
| σ^2^ | 84.57 |  |  |
| τ_00_ _Subject_ | 14.97 |  |  |
| ICC | 0.15 |  |  |
| N_Subject_ | 16 |  |  |
| Observations | 32 |  |  |
| Marginal R^2^/Conditional R^2^ | 0.002 / 0.15 |  |  |
| Model | Ax | Link: log | |
| **Predictors** | **Estimates** | **CI** | **p-value** |
| PM_2.5_ concentration (µg/m^3^) | 0.001 | (0.0001, 0.003) | 0.029* |
| BMI | 0.11 | (-0.02, 0.24) | 0.090 |
| Allergies [Yes] | 0.56 | (-0.89, 2.02) | 0.447 |
| Predicted FEV1 (L/s) | 0.01 | (-0.92, 0.94) | 0.983 |
| Estimated time on subway (hours) | -0.17 | (-0.27, -0.08) | <0.001* |
| **Random effects** |  |  |  |
| σ^2^ | 10.75 |  |  |
| τ_00_ _Subject_ | 14.05 |  |  |
| ICC | 0.57 |  |  |
| N_Subject_ | 17 |  |  |
| Observations | 34 |  |  |
| Marginal R^2^/Conditional R^2^ | 0.07 / 0.60 |  |  |

Subjects were included as the random effect. Fixed effect covariates were visit-type BMI, preexisting allergies (i.e., pollen, dust, mold, etc.), predicted FEV1, PM_2.5_ concentration, total estimated time on subway per week. Wald chi-square tests were employed to determine the significance of the influence of each of the fixed effects variables. * indicates when p-values < 0.05. ICC: intraclass correlation

Table S7 – Logistic mixed effects model of total post-visit symptom scores as a binary outcome

| Model | Total post-visit symptom score with visit | | |
| --- | --- | --- | --- |
| **Predictors** | **Odds ratio** | **CI** | **p-value** |
| Visit [Subway] | 4.64 | (1.12, 19.31) | 0.035* |
| BMI | 0.83 | (0.71, 0.97) | 0.016* |
| Allergies [Yes] | 2.11 | (0.36, 12.35) | 0.406 |
| Predicted FEV1 (L/s) | 1.45 | (0.56, 3.72) | 0.441 |
| Estimated time on subway (hours) | 0.99 | (0.88, 1.12) | 0.918 |
| **Random effects** |  |  |  |
| σ^2^ | 3.29 |  |  |
| τ_00_ _Subject_ | 0.28 |  |  |
| ICC | 0.08 |  |  |
| N_Subject_ | 28 |  |  |
| Observations | 56 |  |  |
| Marginal R^2^/Conditional R^2^ | 0.33 / 0.38 |  |  |
| Model | Total post-visit symptom score with PM_2.5_ concentration | | |
| **Predictors** | **Odds ratio** | **CI** | **p-value** |
| PM_2.5_ concentration (1 sd [µg/m^3^]) | 1.99 | (0.97, 4.10) | 0.061 |
| BMI | 0.83 | (0.71, 0.97) | 0.016* |
| Allergies [Yes] | 2.21 | (0.38, 12.86) | 0.380 |
| Predicted FEV1 (L/s) | 1.52 | (0.60, 3.89) | 0.379 |
| Estimated time on subway (hours) | 1.00 | (0.88, 1.12) | 0.949 |
| **Random effects** |  |  |  |
| σ^2^ | 3.29 |  |  |
| τ_00_ _Subject_ | 0.27 |  |  |
| ICC | 0.08 |  |  |
| N_Subject_ | 28 |  |  |
| Observations | 56 |  |  |
| Marginal R^2^/Conditional R^2^ | 0.30 / 0.36 |  |  |

Total symptom scores greater than the median value (i.e., 15) were considered “high” and the rest were considered “low”. Subjects were included as the random effect. Models are alternatively listed with visit or PM2.5 concentration as a fixed variable. PM2.5 concentrations were scaled to the overall mean and standard deviation. Fixed effect covariates were visit-type BMI, preexisting allergies (i.e., pollen, dust, mold, etc.), predicted FEV1, PM2.5 concentration, total estimated time on subway per week. Wald chi-square tests were employed to determine the significance of the influence of each of the fixed effects variables. * indicates when p-values < 0.05. ICC: intraclass correlation; sd: standard deviation

Table S8 – Logistic mixed effects model of R5-20 as a binary outcome

| Model | R5-20 with visit | | |
| --- | --- | --- | --- |
| **Predictors** | **Odds ratio** | **CI** | **p-value** |
| Visit [Subway] | 0.41 | (0.03, 5.81) | 0.506 |
| BMI | 1.07 | (0.78, 1.48) | 0.662 |
| Allergies [Yes] | 149.38 | (0.27, 822293) | 0.120 |
| Predicted FEV1 (L/s) | 0.02 | (0.0001, 2.47) | 0.111 |
| Estimated time on subway (hours) | 0.96 | (0.75, 1.23) | 0.754 |
| **Random effects** |  |  |  |
| σ^2^ | 3.29 |  |  |
| τ_00_ _Subject_ | 1.25 |  |  |
| ICC | 0.28 |  |  |
| N_Subject_ | 17 |  |  |
| Observations | 34 |  |  |
| Marginal R^2^/Conditional R^2^ | 0.79 / 0.85 |  |  |
| Model | R5-20 with PM_2.5_ concentration | | |
| **Predictors** | **Odds ratio** | **CI** | **p-value** |
| (Intercept) | 95869.2 | (0.001, 1.724 x 10^13^) | 0.237 |
| PM_2.5_ concentration (1 sd [µg/m^3^]) | 0.68 | (0.18, 2.51) | 0.557 |
| BMI | 1.07 | (0.79, 1.46) | 0.664 |
| Allergies [Yes] | 127.48 | (0.30, 53538.48) | 0.116 |
| Predicted FEV1 (L/s) | 0.02 | (0.0002, 2.29) | 0.105 |
| Estimated time on subway (hours) | 0.96 | (0.75, 1.23) | 0.753 |
| **Random effects** |  |  |  |
| σ^2^ | 3.29 |  |  |
| τ_00_ _Subject_ | 1.11 |  |  |
| ICC | 0.25 |  |  |
| N_Subject_ | 17 |  |  |
| Observations | 34 |  |  |
| Marginal R^2^/Conditional R^2^ | 0.79 / 0.84 |  |  |

R5-20 values greater than the median value (i.e., 0.110) were considered “high” and the rest were considered “low”. Subjects were included as the random effect. Models are alternatively listed with visit or PM_2.5_ concentration as a fixed variable. PM_2.5_ concentrations were scaled to the overall mean and standard deviation. Fixed effect covariates were visit-type BMI, preexisting allergies (i.e., pollen, dust, mold, etc.), predicted FEV1, PM_2.5_ concentration, total estimated time on subway per week. Wald chi-square tests were employed to determine the significance of the influence of each of the fixed effects variables. * indicates when p-values < 0.05. ICC: intraclass correlation; sd: standard deviation
